# Drought modulates interactions between arbuscular mycorrhizal fungal diversity and barley genotype diversity

**DOI:** 10.1038/s41598-019-45702-1

**Published:** 2019-07-04

**Authors:** Agnieszka Sendek, Canan Karakoç, Cameron Wagg, Jara Domínguez-Begines, Gabriela Martucci do Couto, Marcel G. A. van der Heijden, Ali Ahmad Naz, Alfred Lochner, Antonis Chatzinotas, Stefan Klotz, Lorena Gómez-Aparicio, Nico Eisenhauer

**Affiliations:** 1 Systematic Botany and Functional Biodiversity, Institute of Biology, Leipzig University, Johannisallee 21-23, 04103 Leipzig, Germany; 2 German Centre for Integrative Biodiversity Research (iDiv) Halle-Jena-Leipzig, Deutscher Platz 5e, 04103 Leipzig, Germany; 3 Department of Community Ecology, UFZ-Helmholtz Centre for Environmental Research, Theodor-Lieser-Strasse 4, 06120 Halle, Germany; 4 Department of Geobotany and Botanical Garden, Martin Luther University of Halle-Wittenberg, Am Kirchweg 2, 06108 Halle, Germany; 5 Department of Environmental Microbiology, UFZ-Helmholtz Centre for Environmental Research, Permoserstrasse 15, 04318 Leipzig, Germany; 6 Department of Evolutionary Biology and Environmental Studies, University of Zürich, Winterthurerstr. 190, Zürich, CH-8057 Switzerland; 7 Fredericton Research and Development Center, Agriculture and Agri-Food Canada, 850 Lincoln Road, Fredericton, New Brunswick, E3B 4Z7 Canada; 8 Institute of Natural Resources and Agrobiology of Seville (IRNAS), CSIC, LINCGlobal, Avenida Reina Mercedes, 10, 41012 Sevilla, Spain; 9 Institute of Biology, Leipzig University, Deutscher Platz 5e, 04103 Leipzig, Germany; 10 Plant-Soil-Interactions, Department of Agroecology and Environment, Agroscope, Reckenholzstrasse 191, 8046 Zurich, Switzerland; 11 Department of Plant and Microbial Biology, University of Zurich, Zollikerstrasse 107, 8008 Zurich, Switzerland; 12 Crop Genetics and Biotechnology Unit, Institute of Crop Science and Resource Conservation, University of Bonn, Katzenburgweg 5, 53115 Bonn, Germany

**Keywords:** Biodiversity, Arbuscular mycorrhiza

## Abstract

Droughts associated with climate change alter ecosystem functions, especially in systems characterized by low biodiversity, such as agricultural fields. Management strategies aimed at buffering climate change effects include the enhancement of intraspecific crop diversity as well as the diversity of beneficial interactions with soil biota, such as arbuscular mycorrhizal fungi (AMF). However, little is known about reciprocal relations of crop and AMF diversity under drought conditions. To explore the interactive effects of plant genotype richness and AMF richness on plant yield under ambient and drought conditions, we established fully crossed diversity gradients in experimental microcosms. We expected highest crop yield and drought tolerance at both high barley and AMF diversity. While barley richness and AMF richness altered the performance of both barley and AMF, they did not mitigate detrimental drought effects on the plant and AMF. Root biomass increased with mycorrhiza colonization rate at high AMF richness and low barley richness. AMF performance increased under higher richness of both barley and AMF. Our findings indicate that antagonistic interactions between barley and AMF may occur under drought conditions, particularly so at higher AMF richness. These results suggest that unexpected alterations of plant-soil biotic interactions could occur under climate change.

## Introduction

Arable land is to date one of the major land use types worldwide^1^. Its management intensity as well as environmental impacts will possibly increase in the future due to the steeply rising human population^2–4^. Simultaneously, low biodiversity resulting from monocropping, makes arable systems prone to stress and disturbances, including intensive droughts related to climate change^5,6^. This is because the adverse effects of global change drivers on ecosystems can be, to some extent, mitigated by high species number and the diversity in species’ functional attributes^7,8^. Despite this knowledge from biodiversity-ecosystem function research, the majority of modern agricultural plantations are designed to intensify production of plant monocultures^9^. Moreover, land-use practices applied to improve plant production in the short term may also lead to a decrease in biodiversity in other ecosystem components^10,11^, such as key soil microbiota that maintain the efficiency in various soil processes and promote plant growth^12,13^. This reduction in soil biodiversity makes agricultural ecosystems even more vulnerable to environmental stress^14,15^.

There is a growing evidence that increasing intraspecific diversity of plants may stabilize community productivity in unfavorable conditions^16,17^. Co-cultivation of multiple varieties of a single crop (*i*.*e*. inter-varietal diversity^18^) was shown to increase the stability of agroecosystems experiencing environmental stress^19,20^. Genetic diversity can influence ecosystem functions directly by modifying population performance or indirectly by affecting the diversity and abundance of species within the same, or other, trophic levels^21–23^. For instance, intraspecific differences among plant individuals in their traits related to stress responses may be a potential mechanism that stabilizes crop yield by increasing the probability that some individuals will resist the perturbation^24^. In the same manner, a greater diversity of mutualistic interactions with various groups of soil organisms may ensure maintenance of soil processes^25–28^.

Among the vast diversity of soil organisms potentially mediating crop productivity, arbuscular mycorrhizal fungi (AMF) are considered to be of particular importance for the sustainable management of agricultural ecosystems^29,30^. The ability of AMF to affect plant-plant relationships is largely based on their role in nutrient uptake^31–33^, increasing resistance against diseases^34^ and improving water-use efficiency^35–37^. Although the majority of terrestrial plants can form an association with AMF, the magnitude and direction of this relationship strongly depend on resource availability^38–41^, as well as on host species^42–44^ and genotype identity^45,46^. Similarly, colonization intensity is often considered to underpin fungi effects on plant growth, total biomass production and its allocation, as well as nutrient dynamics^47–50,51^. For instance, a high percentage of colonized roots is often associated with stronger positive AMF effects on plants^47,48,52^. However, relationships between AMF colonization and plant growth were also reported to differ across the studies (*e*.*g*.^53–55^), suggesting that effects of root colonization may also depend on other drivers like species identity or environmental conditions^48^. Under optimal environmental conditions (*e*.*g*. high water and nutrient availability), interactions with AMF may be of lower importance for the plant host^40,56^ or even lead to antagonistic relations^57^. This can be particularly the case for plants characterized by expansive and fibrous root systems that depend less on the assistance of AMF^58^. As a consequence, the importance of AMF for traditional crop production is questioned^59^. Relations between AMF and a host plant may, however, change under limiting resource conditions^38,44^, where a diverse AMF community may act as an insurance for resource availability^33,60^. Higher diversity of AMF can increase the number of mutualistic interactions and relax interspecific plant competition^33,61^. The strength of these positive effects, however, differs across studies^62,63^ and depends on the identity and traits of the plant-fungal partners^64^. For instance, modern crop cultivars may form weaker associations with AMF than their wild relatives^40,65–67^ due to differences in root morphology and amount and quality of root deposits^68^. Consequently, mixtures of plant varieties differing in root traits may not only be more resistant to drought^20^, but may also maintain a higher abundance and diversity of AMF^69–71^, as it was previously shown for bacterial communities^72,73^.

Although the importance of both intraspecific plant diversity and AMF diversity for ecosystem functioning has often been highlighted^17,70,74^, how these two interactively influence each other’s performance remains unstudied. With the increasing awareness of the important role of interactions across trophic levels^75,76^, there is a need for studies to adopt a whole-ecosystem perspective in biodiversity-ecosystem function research^77–80^. A better understanding of the impact of diversity at different levels of ecosystem organization can help improve the productivity, health, and sustainability of ecosystems. This is of particular importance for agricultural systems^67,81^ and may provide promising management options to mitigate the consequences of climate change^6^.

**Figure 1:**
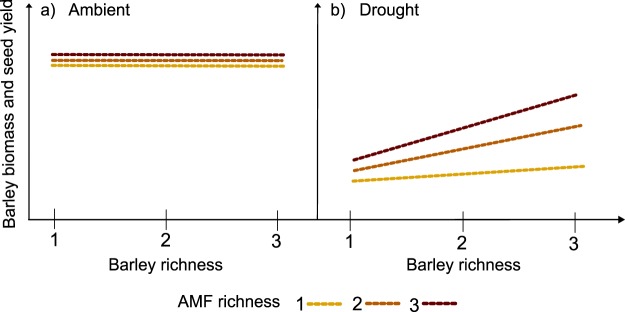
Graphical representation of the main hypothesis. Panel (a) shows expected effects of barley richness and AMF richness on barley biomass and seed yield in ambient conditions. Panel (b) shows expected effects of barley richness and AMF richness on the barley biomass and seed yield in drought conditions.

Here, we investigated the joint effects of genotype diversity of crop plants and AMF species diversity on yield under ambient water and drought conditions. As model species, we selected three barley genotypes systematically varying in root system size and three common AMF isolates occurring in arable fields. We hypothesized (1) that the drought-induced reduction of barley seed production will be mitigated by barley diversity, AMF richness, and their interaction, so that the highest yield under drought conditions will be obtained in the combination of highest barley and AMF richness (Fig. 1). Moreover, we expected (2) that interactive effects of barley richness and AMF richness on barley and AMF performance will be mediated by the mycorrhiza colonization frequency of roots.

## Materials and Methods

### Plant and fungi material

We used three barley genotypes and three species of arbuscular mycorrhizal fungi (AMF) to generate our above- and below-ground diversity gradients, respectively. The three barley genotypes included the spring barley cultivar ‘Scarlett’ (*Hordeum vulgare*), the wild barley accession ISR42-8 (*Hordeum vulgare* ssp. *spontaneum*), and the introgression line S42IL-176. The introgression line S42IL-176 was created by crossing Scarlett and ISR42-8 and *via* successive backcrossing with Scarlett as recurrent parent, so that it carried a QTL allele of wild origin for root biomass in the ‘Scarlett’ background. We chose these three barley genotypes for their gradient in root system size: from the ‘Scarlett’ with the lowest root biomass to wild barley ISR42-8 with the highest root biomass^82^. Extensive root systems are primarily associated with drought avoidance and can improve plant performance under water deficiency^83^, and consequently maintain grain yield under drought^84^. We did not expect any other systematic difference in relation to drought resistance among the examined plants. Beside root size, seed production also differed among the used genotypes. The ‘Scarlett’ accession and the introgression line S42IL-176 represent the high-yielding cultivated germplasms, which were achieved by domestication and intensive breeding^82^. On the contrary, ISR42-8 is a pre-domestication barley accession, which is unlikely to increase grain yield of mixed crop stands in ambient conditions. However, it is expected to stabilize yield of mixed crop stands under drought (Fig. 1).

Belowground diversity was represented by three common species of AMF fungi: *Rhizoglomus intraradices* (isolate BEG 21^85^), *Claroideoglomus claroideum* (isolate JJ 132, N.C.Schenck. & G.S. Sm., C. Walker & A. Schüssler formerly named *Glomus claroideum*^12^) and *Funneliformis mosseae* (isolate BEG 161, T.H. Nicolson & Gerd.C. Walker & A. Schüssler, formerly named *Glomus mosseae*^12^). These species are widespread in various plant communities^86,87^ and abundant in agricultural ecosystems^64,88^. Previous studies demonstrated that they differ in their nutrient uptake mechanisms, growth rates duration of growth phase, carrying capacities and colonization plateau values, as well as vesicle production^88,89^. These differences may facilitate coexistence, and potentially increase their productivity in mixed cultures compared to the monocultures^64,89^. For instance, *F*. *mosseae* is a fast and efficient colonizer, which often dominates mixed cultures^89^. Still, it declines after reaching its colonization plateau at the early stages of plant growth^89^. By contrast, both *C*. *claroideum* and *R*. *intraradices* colonize roots slower and at a lower rate (but see^33^). Moreover, they are less dominant, and their mixtures show complementarity related to P uptake^89^.

Despite the differences in foraging strategies, colonization speed and efficiency^33,64,90^, all selected fungi species are considered effective symbionts, significantly promoting plant growth and phosphorus uptake (*i*.*e*. mostly functioning as plant mutualists)^89,90^. All inocula used in the experiment were obtained from the Swiss collection of Arbuscular Mycorrhizal Fungi (www.agroscope.ch/saf).

### Experimental setup

Richness gradients of 1, 2, or 3 barley genotypes and AMF species were factorially combined. The mixtures of two barley genotypes or two AMF species included all pairwise combinations (see Table 1). In order to balance replication for each barley genotype and AMF richness level, the three barley genotypes or AMF species richness levels crossed with the single or paired barley/AMF richness combinations, respectively, were replicated three times (Table 1). The combination of all three barley genotypes and AMF species was replicated nine times (Table 1). This arrangement was then duplicated under both ambient and drought treatments for a total of 162 microcosms, located in a single growth chamber. Notably, our experiment was specifically designed to investigate the role of biodiversity. Because of that, it cannot properly address questions regarding genotype/species identity and composition. We are aware of the fact that both fungi and plant identity may play an important role in relations between them^91^; therefore, we encourage future studies to focus on identity and community composition effects.

**Table 1:** Design of the experimental setup, representing the number of microcosms assigned to each combination of richness level of barley genotypes (X, Y, and Z) and AMF species (A, B, and C).

		AMF richness						
		A	B	C	AB	AC	BC	ABC
Barley richness	**X**	1	1	1	1	1	1	3
	**Y**	1	1	1	1	1	1	3
	**Z**	1	1	1	1	1	1	3
	**XY**	1	1	1	1	1	1	3
	**XZ**	1	1	1	1	1	1	3
	**YZ**	1	1	1	1	1	1	3
	**XYZ**	3	3	3	3	3	3	9

Blocks of the same richness level included always nine microcosms and are indicated by numbers. The presented setup was replicated for drought and ambient treatments.

Microcosms were made of PVC pipes (inner diameter 10 cm, depth 20 cm) sealed with a 15 µm-mesh at the bottom to allow water drainage. Microcosms were filled with 1.4 L of soil obtained from the UFZ- experimental field station in Bad Lauchstädt, Germany^92,93^; see Table S1 for soil C, N, pH values). The soil was sieved and autoclaved two times at 121 °C for 20 min. We acknowledge that relationships between plants and soil biota are manifold and still not well understood^94,95^. Therefore, we focused on manipulating only one group of soil organisms, *i*.*e*. AMF, while maintaining a close similarity to natural systems. To achieve that, we inoculated pots with a prepared microbial soil wash. Each microcosm was inoculated with 15 ml of the wash obtained by suspending 1 kg of experimental soil with 1 L sterile, distilled water, and sieving with descending mesh sizes (from 500 mm to 15 µm). Sieving is a commonly used method to sort guilds of soil organisms^96–98^ and as such can be applied to decrease the abundance or to fully remove spores of wild AMF species. To establish mycorrhizal treatments in each pot, we mixed 20 g of AMF inocula with the soil. We kept the total weight of AMF inocula constant for each treatment, hence mixtures of AMF inocula were added to pots in equal parts, as it was done in a replacement series in plant diversity experiment^99^. Inoculated pots were thereafter assigned to the barley diversity treatment and finally to the drought treatment. To sterilize seeds, we washed them for 1 min in 70% ethanol solution in 50 ml falcon tubes and removed ethanol residues with MiliQ water. Hereafter seeds were washed in 50% household bleach for 10 minutes. Lastly, they were washed with MiliQ water five times to remove all the bleach residuals. Washed seeds were planted in the 1% agar plates under sterile conditions. Plates were then moved to a chamber (light intensity PAR: 47.5 µmol.m^−2^s^−1^, temperature: 20 °C) for germination. Three individuals of five days-old barley seedlings were transplanted into pots. In microcosms with one barley genotype (monocultures), three individuals of each genotype were planted. In microcosms with three barley genotypes, one individual of each of the three genotypes were planted. In microcosms with two barley genotypes, we kept the number of three plant individuals per microcosm constant by planting one individual of one genotype and two individuals of the other genotype. We randomly selected the genotypes which were represented by two individuals.

The experiment was conducted in a growth chamber with a day/night regime of 16/8 h, temperature of 20/15 °C, and light intensity (PAR) was set at 200 μmol m^−2^s^−1^. Microcosms were randomized every three weeks within the chamber. We estimated a sufficient amount of water for the ambient and drought treatments, on the basis of a preliminary trial (Supplementary Note S1). Each pot was watered three times a week with differing amounts of water (20, 30, 40, 50 ml; Table S2). We applied the treatment immediately after the seedlings had been planted into the pots. Microcosms of the drought treatment received 50% of the amount of water used in ambient treatment throughout the experiments, resulting in substantial differences in water availability. Milli-Q water (MiliQ Thermo Scientific™ Barnstead™ GenPure™), and sterile equipment were used to avoid contamination.

After 17 weeks of growth, the experiment was terminated. Seeds were harvested along with the shoot biomass. To estimate root biomass and its distribution, soil and roots were removed from the PVC pipes intact, and the soil core was separated into three 5 cm-layers, referred to as shallowest (0–5 cm), intermediate (5–10 cm) and deepest (10–15 cm). Approximately 0.15 g of fine roots from the shallowest subsample were extracted, washed, and preserved in 70% ethanol for estimation of AMF colonization frequency. Roots were stained with the ink-vinegar method^100^. We modified the duration of staining to 1.5 h in KOH (room temperature) and 1.5 h in ink (80 °C). AMF colonization was estimated for 30 root fragments of 1 cm length and mounted in lactic acid (60%). The root fragments were scored for the presence of arbuscules, vesicles, and intraradical hyphae to calculate the frequency of mycorrhiza colonization, also the arbuscule and vesicle abundance in root system according to Trouvelot’s method^101^. The remaining roots from all soil depths were washed and dried along with the shoot and seeds at 75 °C for 24 h (constant weight) to determine the shoot and root biomass, as well as the crop yield. Root biomass distribution was calculated as the proportion of root biomass from each layer to the total root biomass.

### Data analysis

We tested the effects of drought, barley genotype richness, AMF species richness, and their interaction on the performance of both plants and AMF. We used seed production (crop yield), shoot biomass, root biomass, and root biomass distribution into soil layers as indicators of plant performance. The frequency of mycorrhizal colonization and the abundance of arbuscules and vesicles in roots were used as indicators of AMF performance. As we expected other treatment effects to be dependent on the frequency of mycorrhiza root colonization, we included mycorrhiza colonization frequency as an explanatory variable in the statistical analyses. Intensity of root colonization by AMF is considered a legitimate measure of the importance of fungi as a modifier of plant-associated C allocation, *i*.*e*. recognized as a predictor of plant functioning^47^. Similarly, meta-analyses acknowledge the effect of root colonization on plant growth, biomass, root:shoot ratio, and P content, by using it as a predictor of respective effect sizes^48,49^.

Although pairwise Pearson’s correlation coefficients of predictor variables did not exceed the threshold of |r| = 0.7, suggesting that collinearity among variables does not influence statistical inference^102^, we interpreted obtained results with a great care. Additionally, we ensured that the abundances of arbuscules and vesicles are not strongly correlated (Fig. S1). For each of the response variables, we carried out a multiple linear regression with normal error distribution. The only exception was mycorrhiza colonization frequency, which was analyzed using a logistic regression with quasibinomial error distribution and a logit link. Whenever assumptions of normality and heteroscedasticity were violated, we applied logarithmic or Box-Cox transformation. We treated the richness of barley genotypes and AMF species as linear variables. However, to test if observed patterns remain unchanged, we repeated the analysis, treating diversity of barley and AMF as factors (Tables S3 and S4).

To test if effects of drought and barley/AMF richness on plant performance can be explained by mycorrhiza colonization frequency, we used structural equation models^103^. The exogenous variables encompassed barley genotype diversity, AMF diversity, and drought. We fitted separate models for seed mass, shoot biomass, and root biomass, which along with AMF colonization frequency were treated as endogenous variables. After fitting the full model, we performed a stepwise removal of non-significant and weak relationships to obtain the most parsimonious model. Selection was based on the decrease in the Bayesian information criterion (BIC) and non-significant chi-square tests (p > 0.05)^103^.

All data analysis and visualizations were conducted using R version 3.2.3^104^. Regression models were carried out using ‘lm’ function or ‘glm’ functions of the package ‘stats’^104^. To transform response variables, we used the ‘boxCox’ function of the ‘car’ package^105^. To fit path analysis, we used the ‘sem’ function of the ‘lavaan’ package^106^. Plots were made with the help of packages ‘visreg’^107^ and ‘ggplot2’^108^. The conceptual figure presenting the hypothesis (Fig. 1) was created using the open source vector graphics software Inkscape (Version 0.91)^109^.

## Results

### Barley performance

**Figure 2:**
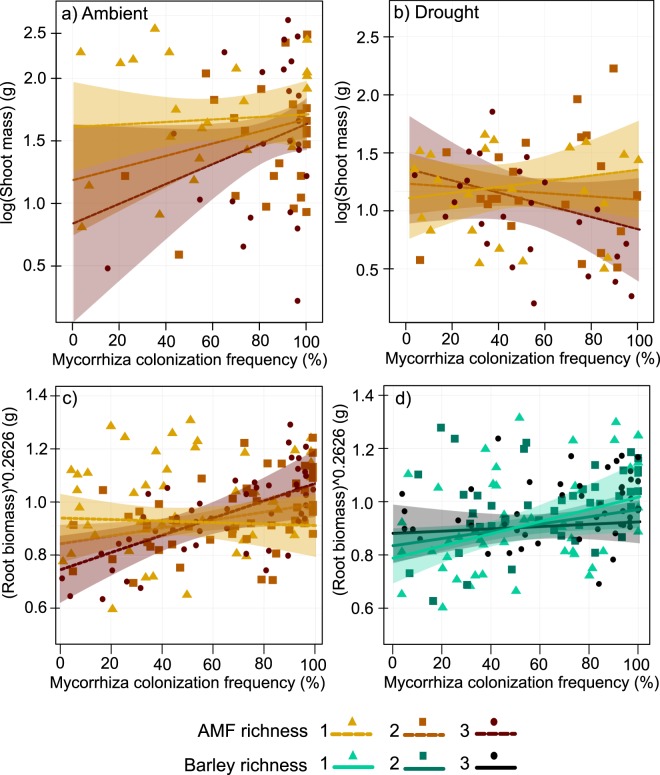
Interactive effects of treatments on plant performance. Panels (a,b) show the effect of the drought treatments, AMF richness, and mycorrhiza colonization frequency on shoot biomass. Panel (c) shows the effect of mycorrhiza colonization frequency of roots and AMF richness on root biomass. Panel (d) shows the effect of mycorrhiza colonization frequency of roots and barley richness on root biomass. Lines represent fitted values with 95% confidence intervals.

Seed mass (−36%), shoot biomass (−40%), and root biomass (−42%) decreased under the drought treatment (Table 2). Drought also affected the root allocation pattern across different soil depths with higher root biomass investment into the deepest soil layer compared to the shallowest one (Table 2). AMF richness and AMF colonization frequency altered the effect of drought only in case of shoot biomass (Table 2, Fig. 2a,b). In ambient water conditions, shoot biomass was lowest at high AMF richness, specifically when mycorrhiza colonization frequency was low (Table 2, Fig. 2a). On the contrary, high mycorrhiza colonization frequency of roots increased the shoot biomass of barley and reduced the effect of AMF richness (Table 2, Fig. 2a). In drought conditions, the positive effect of high mycorrhizal colonization frequency on plant shoot biomass was maintained only in AMF monocultures (Table 2, Fig. 2b).

**Table 2:** Test statistics of the linear models used to explore effects of drought and diversity treatments on barley performance.

Explanatory variables	Seed mass			Shoot biomass		Root biomass		Shallowest root-layer		Intermediate root-layer		Deepest root layer	
	D.f.	F	p	F	p	F	p	F	p	F	p	F	p
Drought	1	11.33	**0**.**001**	35.37	<**0**.**001**	41.12	<**0**.**001**	12.86	**0**.**001**	0.14	0.70	13.37	**<0**.**001**
Barley richness	1	0.50	0.48	0.97	0.33	9.61	**0**.**002**	2.84	0.09	0.34	0.55	2.04	0.16
AMF richness	1	1.90	0.17	4.25	**0**.**041**	4.04	**0**.**046**	2.83	0.09	2.62	0.10	8.89	**0**.**003**
Colonization frequency	1	0.02	0.90	0.33	0.56	10.59	**0**.**001**	0.81	0.37	1.76	0.18	3.68	0.06
Drought: barley richness	1	0.34	0.56	2.70	0.10	3.24	0.07	1.12	0.29	0.21	0.64	0.69	0.41
Drought: AMF richness	1	0.00	0.95	0.04	0.84	0.87	0.35	2.69	0.10	0.49	0.48	1.69	0.20
Drought: colonization frequency	1	0.73	0.40	2.91	0.09	1.55	0.21	2.75	0.09	0.27	0.27	2.08	0.15
Barley richness: AMF richness	1	0.01	0.92	0.87	0.35	0.58	0.45	5.34	**0**.**022**	0.00	0.99	6.43	**0**.**012**
Barley richness: colonization frequency	1	1.00	0.32	2.78	0.10	5.85	**0**.**017**	1.38	0.24	0.82	0.36	0.42	0.52
AMF richness: colonization frequency	1	0.23	0.63	0.09	0.76	7.88	**0**.**006**	6.87	**0**.**009**	14.10	<**0**.**001**	0.05	0.83
Drought: barley richness: AMF richness	1	0.38	0.54	0.97	0.33	0.13	0.72	1.04	0.31	0.51	0.47	0.37	0.54
Drought: barley richness: colonization frequency	1	2.90	0.09	1.26	0.26	0.02	0.89	0.55	0.46	0.50	0.48	0.10	0.76
Drought: AMF richness: colonization frequency	1	2.59	0.11	4.53	**0**.**035**	0.04	0.84	0.00	0.97	0.57	0.45	0.32	0.57
Barley richness: AMF richness: colonization frequency	1	0.93	0.34	0.62	0.43	1.95	0.16	2.50	0.11	0.89	0.34	1.14	0.29
Adjusted R^2^	0.0567			0.2353		0.3303		0.1656		0.0584		0.1548	
No. observations	149			143		150		150		150		150	

The table shows degrees of freedom (D.f.), values of F statistic (F) and p values (p) for each main effect and interactions up to the 3rd order. Degrees of freedom (D.f.), values of F statistic (F) and p values (p) are presented for each main effect and interactions up to the 3rd order. Significant p values (p < 0.05) are highlighted in bold. Root layers (shallowest, intermediate, deepest) represent root biomass allocation to soil layers. Barley and AMF richness are treated as linear terms.

Root biomass increased with mycorrhiza colonization frequency independent from the water conditions (Table 2, Fig. 2c,d). This positive relationship was, however, only observable in treatments with more than one AMF species (Table 2, Fig. 2c). Contrastingly, the root biomass increase in the high mycorrhiza colonization frequency was strongest in the barley monocultures (Table 2, Fig. 2d). High plant genotype richness and AMF richness reduced the root allocation to the shallowest soil layer (Table 2, Fig. S2a). The effect of AMF was, however, reversed at low mycorrhiza colonization frequency (Table 2, Fig. S2b). Root allocation to intermediate and deepest soil layers increased with high AMF richness (Table 2, Fig. S2c,d). Additionally, this pattern was strengthened in the midmost layer with mycorrhiza colonization frequency (Table 2, Fig. S2d). Lastly, root allocation to the deepest soil layer also increased with plant genotype richness (Table 2, Fig. S2c).

### AMF performance

**Figure 3:**
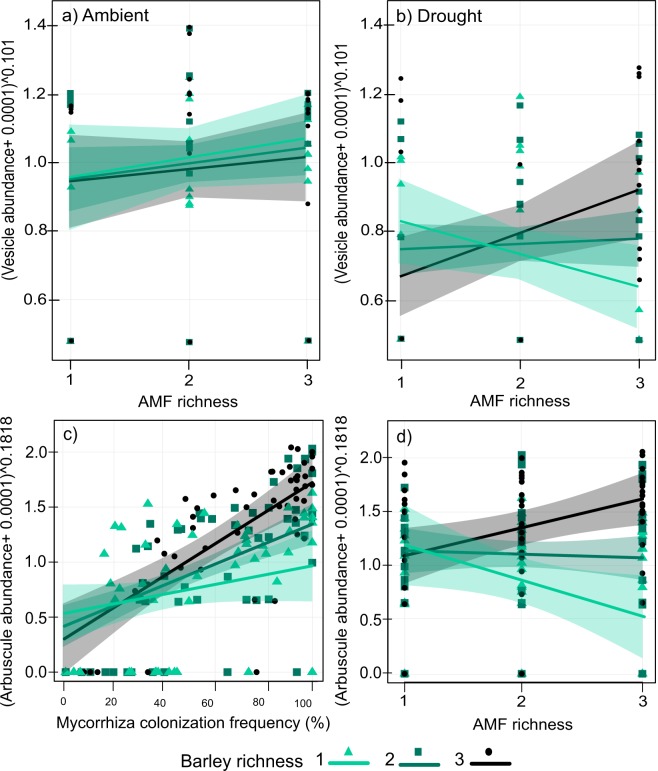
Interactive effects of treatments on AMF performance. Panels (a,b) show the effect of the drought treatments, barley richness, and AMF richness on vesicle abundance. Panel (c) shows the effect of barley richness and mycorrhiza colonization frequency of roots on arbuscule abundance. Panel (d) shows the effect of barley richness and AMF richness on arbuscule abundance. Lines represent fitted values with 95% confidence intervals.

Drought decreased all indicators of AMF performance, *i*.*e*. mycorrhiza colonization frequency [−31%], and the abundance of arbuscules [−58%] and vesicles [−64%] (Table 3). Vesicle abundance was affected by the interaction of plant genotype richness, AMF richness, and drought treatment (Table 3, Fig. 3a,b). In ambient conditions, the abundance of vesicles increased with increasing AMF richness (Table 3, Fig. 3a). In drought conditions, however, this effect gradually decreased with decreasing plant genotype richness (Table 3, Fig. 3b).

**Table 3:** Test statistics of the linear models used to explore effects of drought and diversity treatments on AMF performance.

Explanatory variables		Arbuscule abundance		Vesicle abundance		Mycorrhiza frequency	
	D.f.	F	p	F	p	F	p
Drought	1	27.23	<**0**.**001**	17.81	<**0**.**001**	25.09	<**0**.**001**
Barley richness	1	34.05	<**0**.**001**	7.84	**0**.**006**	7.99	**0**.**005**
AMF richness	1	20.42	<**0**.**001**	34.72	<**0**.**001**	8.86	**0**.**003**
Colonization frequency	1	110.00	<**0**.**001**	139.54	<**0**.**001**	X	X
Drought: barley richness	1	0.23	0.63	1.06	0.30	0.28	0.60
Drought: AMF richness	1	0.10	0.75	0.99	0.32	0.95	0.33
Drought: colonization frequency	1	1.19	0.28	0.50	0.48	X	X
Barley richness: AMF richness	1	4.06	**0**.**046**	2.60	0.11	2.41	0.12
Barley richness: colonization frequency	1	5.12	**0**.**025**	1.39	0.24	X	X
AMF richness: colonization frequency	1	0.18	0.68	1.38	0.24	X	X
Drought: barley richness: AMF richness	1	3.18	0.08	5.97	**0**.**016**	0.00	0.97
Drought: barley richness: Colonization frequency	1	1.94	0.17	0.66	0.42	X	X
Drought: AMF richness: colonization frequency	1	1.74	0.19	0.01	0.94	X	X
Barley richness: AMF richness: colonization frequency	1	1.68	0.20	4.56	**0**.**034**	X	X
Adjusted R^2^	0.5667			0.5664		0.1973	
No. observations	158			158		158	

The table shows degrees of freedom (D.f.), values of F statistic (F) and p values (p) for each main effect and interactions up to the 3rd order. Significant p values (p < 0.05) are highlighted in bold. Symbol X stands for the significance of the main effects and interactions which could not be calculated when mycorrhiza colonization frequency was used as the response variable. Barley and AMF richness are treated as linear terms.

Other effects of barley richness and AMF richness on AMF performance were not significantly affected by drought. Mycorrhiza colonization frequency of roots was positively affected by the richness of barley genotypes and the richness of AMF species (Table 3). Increasing barley richness strengthened the positive effect of mycorrhiza colonization frequency on the abundance of arbuscules (Table 3, Fig. 3c) and reversed the effect of increasing AMF richness from negative in barley monocultures to strongly positive at the highest level of barley richness (Table 3, Fig. 3d). The abundance of the vesicles also increased with increasing mycorrhiza colonization frequency of roots (Table 3, Fig. S3a–c). However, at low mycorrhiza colonization frequency, barley richness had a strong negative effect on the abundance of vesicles, especially at higher AMF richness (Table 3, Fig. S3a–c).

### Mycorrhiza colonization frequency as a potential mediator of treatment effects

**Figure 4:**
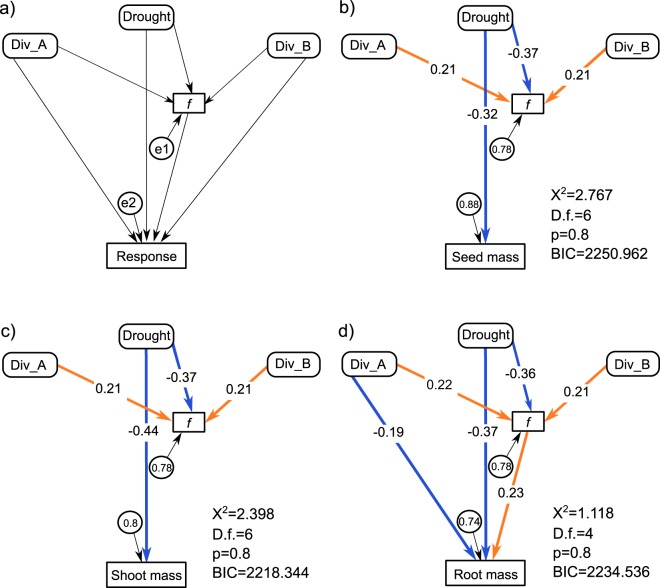
Direct and indirect (through changes of mycorrhiza colonization ‘*f’*) effects of drought, barley richness (Div_B), and AMF richness (Div_A) on measures of barley and AMF performance indicated by structural equation models. Panel (a) shows the initial model, while panels (b-d) show the most parsimonious models for seed mass, shoot biomass, root biomass, respectively. Values of χ^2^ and BIC as well as degrees of freedom (D.f.) and p values (p) related to the models are presented at the right bottom corner of each panel. Endogenous variables are displayed in squares, while exogenous variables are given in rounded squares. Standard errors (e1, e2) are given in circles next to their corresponding variables. Significant relationships are illustrated by arrows. Numbers on arrows represent standardized path coefficients, while their color indicates the direction of relationships (orange – positive, blue – negative).

The structural equation models explained 10% and 11% of the variance in seed mass and shoot biomass, respectively. For both variables, the most parsimonious models (Table S5, Fig. 4b,c) retained only the direct negative effect of drought on the response variables and on mycorrhiza colonization frequency, as well as the positive effects of barley richness and AMF richness on mycorrhiza colonization frequency (Table S5, Fig. 4b,c). In the case of root biomass, the most parsimonious model explained 26% of its variance (Table S5, Fig. 4d). It included a direct negative effect of drought on root biomass, as well as an indirect negative effect by reducing mycorrhiza colonization frequency of roots, which was positively related to root biomass. Both barley richness and AMF richness had a positive indirect effect on root biomass by increasing mycorrhiza colonization frequency of roots. AMF richness had, however, a direct negative effect on root biomass that was not explained by mycorrhiza colonization frequency.

## Discussion

The results of the present study could not confirm our expectation regarding the mitigation of a negative drought effect on the crop yield by the positive interactions between AMF diversity and barley genotypic diversity. Barley performance substantially decreased in response to drought, as indicated by reduced seed yield, shoot and root biomass. Although we did not observe any evidence of an overall higher investment into roots that might be expected under drought conditions^110,111^ (but see^112^), more root biomass was allocated to deeper soil layers. Observed changes in root allocation may have been caused by a reduced strength of the drought treatment on the soil moisture in deeper soil depths^113^. Plant responses to drought have been shown to be highly complex and depend on multiple processes and pathways^114^. Here, we focused on plant biomass-related responses of barley genotypes that represent systematic differences in roots size. In addition, physiological attributes related to *e*.*g*. transpiration may be also addressed in similar settings (*e*.*g*.^115^) in future studies and may provide important complementary information on interactive effects of drought with below- and aboveground diversities.

Importantly and in contrast to our expectations, mycorrhiza colonization frequency did not mediate the negative effect of drought on shoot biomass and seed mass. Results indicate that the high mycorrhiza colonization frequency does not necessarily buffer the effects of water shortage and may not be sufficient to mitigate the effects of drought events caused by climate change. This finding is in contrast to some earlier studies that reported positive effects of AMF species on plant responses to drought^116^. The most prominent empirical evidences were delivered by studies using mycorrhiza accessions pre-adapted to water shortage (*e*.*g*. inocula obtained from dry regions^117,118^. In this study, we aimed to represent commonly occurring conditions, where plant composition is actively managed by a farmer, while the composition of AMF fungi is not manipulated intentionally. Therefore, we used widespread AMF species, which frequently occur in agricultural fields^64,88^. Although in intensively managed agricultural systems, AMF richness is often strongly reduced^12,119,120^, the number of fungi species used in this study can be considered low in comparison to the natural systems (*e*.*g*.^39^).

AMF colonization frequency was found to underlie many of the observed effects. For instance, the differences in shoot biomass, observed between AMF richness levels under ambient water conditions, occurred only when root colonization frequency was low. One potential explanation can be related to a higher initial carbon investment of plants into the development of mycorrhizal structures, suggesting that observed stages of mycorrhizal networks are not developed enough to compensate for the carbon investment by the plant^121^. This potential effect might have even been intensified by the experimental conditions. Relatively low light intensity, for instance, might have limited the efficiency of photosynthesis and thus carbon supply to the plant-AMF network. Moreover, differences in colonization frequency may depend on AMF species identity (Fig. S4), especially as the used species are known to differ in colonization rate and efficiency^88,89^. The same fungi inoculates that we applied in our study were previously used by Wagg *et al*.^33^, who observed that *G*. *intraradices* had the highest colonization rates in roots, followed by *G*. *claroideum* and *G*. *mosseae*. However, since different plant species and treatments were used in these two studies, we cannot directly compare our findings with those of Wagg *et al*.^33^, despite the similarity of colonization patterns (Fig. S4). In this study, we did not determine the identity of particular fungi species in mixtures. A more detailed explanation of observed patterns, *i*.*e*., colonization frequency of particular species, would require future studies to focus not only on diversity effects, but also on identity effects of different AMF species. Furthermore, we conducted our sampling at the end of the barley growth period, when AMF colonization tends to be lower^122^. Recent studies show the importance of temporal dependencies of AMF-plant interactions, as reflected by the differences in colonization rate and plant metabolism dynamics^122–124^. Future studies should thus explore the temporal dynamics of mutualistic relationships and their context-dependency in agricultural systems.

Although obtained results did not confirm our main hypothesis of buffering effects of biodiversity, effects of drought on shoot biomass differed across AMF richness levels and with mycorrhiza colonization frequency. In ambient water conditions, we observed a slight increase in shoot biomass with an increase of mycorrhiza colonization frequency, suggesting the prevalence of mutualistic relations between plants and AMF^89^. Under drought conditions, this positive relationship remained only in AMF monocultures, while higher AMF richness led to a decrease of shoot biomass. This finding suggests that while a single AMF species was sufficient to increase plant biomass under water stress in our experimental set up, higher AMF richness reversed this effect. Several studies found that the presence of efficient AMF species plays a more important role than AMF diversity^33,41,125,126^. Moreover, a higher number of AMF species may not necessarily lead to greater complementarity, as it can enhance competitive interactions^89,127–130^. This, in turn, can reduce plant benefits from AMF colonization^41,130^. Furthermore, as different AMF species may vary in their carbon demands, it is also possible that more ‘assimilate-expensive’ AMF species included in the mixture caused plant growth depression^126,131,132^. Although environmental mechanisms can also underlie the observed decrease of plant biomass^57^, it is unclear whether it can be triggered by water stress. The study of Querejeta *et al*.^133^ showed that under drought conditions fungi benefit from water transport from plant roots to the mycelium; however, potential consequences of this effect for plants are ambiguous.

In contrast to shoot biomass, the effects of both AMF richness and barley richness on root biomass did not differ between ambient and drought conditions. Moreover, AMF richness and barley richness had contrasting effects on these variables. Elevated root biomass was related to higher mycorrhiza colonization frequency, which can be interpreted as a reaction to stronger belowground competition^134,135^, especially as we did not observe a corresponding pattern for shoot biomass. The opposing effect of barley richness can be caused by differences in root length among the barley genotypes^82^, which allowed more efficient resource allocation and reduced the root biomass. Besides the overall increase in root biomass, higher AMF richness increased root biomass investment into deeper soil layers, especially at higher AMF colonization frequency. This effect is similar to the one caused by drought stress and suggests that plants needed to extend their root systems, particularly to lower soil depths, to obtain more resources^136,137^. These two findings can result from a dominance of an antagonistic relationship between AMF and plants, observed mostly in nutrient-rich soil^44,57,126,138^. Furthermore, while higher AMF diversity increased root biomass *via* elevated root colonization intensity, our structural equation model indicated the presence of a concurrent negative effect (direct path in the SEM). This implies that interactions between plants and AMF may have multiple, and sometimes opposing effects on plant performance^44,139^. These relations depend, among other factors, on the diversity of plant and belowground communities^33,42,140^ as well as soil nutrient status, and disentangling them still requires scientific attention.

In comparison to the host plants, the impact of abiotic stress on AMF performance is under-examined^141^. For instance, observed effects of drought on AMF colonization may vary from positive to negative^36,142^. In our study, drought caused a decrease in colonization frequency and, correspondingly, in formation of arbuscules and vesicles. Similar responses to drought were also reported in previous studies^43,143–145^ indicating impaired AMF functioning, and indirectly reduced plant performance^37^. Similar to the plant performance, the drought effect on mycorrhiza performance depended on barley diversity. For example, under drought stress, the abundance of vesicles was positively related to AMF richness only at high barley richness. Vesicles serve mostly as storage of photosynthesis products^32^ or propagules^146^. Their abundance can thus be expected to decrease under stress conditions, *e*.*g*. limited water availability^43^. In our experiment, high barley richness translated into a higher diversity of root traits (*i*.*e*. root length and volume) which may provide different niches for different AMF species and thus allow greater complementarity between them^91,147,148^. Additionally, it has been proposed that due to a long history of domestication, modern crops are less susceptible to microbial partners than their wild relatives^66,67,149^. Here we show that higher richness of wild and domesticated barley cultivars may have a potential to improve the performance of AMF.

Contrastingly, vesicles can also be interpreted as resting structures occurring in dead or stressed roots^150,151^. Notably, Cabello^150^ found that the abundance of vesicles is negatively correlated with the abundance of arbuscules under pollution stress. According to Martínez-García *et al*.^43^, increasing numbers of arbuscules at the expense of vesicles represent an intensification of interactions with the host plant in response to drought. In our study, however, numbers of vesicles and arbuscules were weakly, positively correlated (compare Fig. S1), providing more support for the former interpretation of beneficial effects of root trait diversity on AMF performance.

Finally, observed patterns of vesicle abundance may also partly be attributed to the identity effects of the AMF species. For instance, *Rhizoglomus intraradices* can produce substantially more vesicles than other common AMF species^89^. On the contrary, numbers of vesicles produced by *G*. *mosseae* are exceptionally low^89^. Despite structural similarities, even closely related AMF species may differ greatly in their functional characteristics^42,139^, and also in colonization pace or strength of their associations with plant genotypes^89^. While the identity effects of different AMF species and barley genotypes is beyond the scope of our study (see materials and methods), future studies may be able to relate AMF identity effects to their functional traits^152–154^.

In contrast to the vesicle abundance, the effects of both AMF richness and barley richness on the arbuscule abundance did not differ between ambient and drought conditions. We found that the arbuscule abundance, similarly to the AMF colonization rate, increased with barley diversity. These findings are in agreement with other studies where the abundance of arbuscules decreased with decreasing mycorrhiza colonization rate (*e*.*g*.^145^; but see^37^). Arbuscules are considered to be the major sites of exchange between the fungi and their host plant^32,42^; their high abundance thus indicates strong interactions between plants and AMF. Interestingly, the observed positive effect of barley richness on the abundance of arbuscules, may provide further support for our conclusions regarding the positive influence of plant genotypic richness on AMF. Our results confirm the findings of other studies, which reported a high fungal selectivity, also for plant intraspecific diversity^66,67,155^. Notably, the effect of AMF diversity on the arbuscule abundance varied from positive at high barley genotypic richness to negative in barley monocultures. This not only confirms that relations between particular AMF taxa may be adverse^89,156^, but also indicates that the higher diversity of root traits provided by barley genotypic diversity begets higher belowground diversity, as observed in other studies^156–159^.

We found an evidence for the complex and context-dependent interactive effects of AMF and barley richness. Our results indicate that the genotypic richness of barley can improve the performance of the associated AMF community and perhaps even facilitates the co-existence of a higher number of AMF species. The role of AMF in ecosystems exceeds direct interactions with plants and includes the provisioning of key ecosystem services in sustainable agriculture like soil aggregation and carbon sequestration^30,160^. Here, we demonstrated that an increase in plant genotype richness improves the performance of the fungal partner in ambient conditions and, to some extent, under drought conditions. Furthermore, our study illustrates the complexity of the AMF richness effects on plant performance. Our results showed that the mycorrhiza colonization frequency of roots can mediate root biomass production, while this may also diminish under drought stress. This result further exemplifies how biotic interactions and biodiversity-ecosystem function relationships depend on environmental conditions^161^. Trait-based approaches with a temporal dimension are necessary to mechanistically understand identity effects, mycorrhizal functions and their temporal aspects^33,61,127^. Furthermore, a better understanding on plant-soil relations in a climate change context requires precise estimation of drought stress in accordance with field observations and climate change scenarios. Such studies will help understand the role of genotypic crop diversity in agricultural settings better. Also, understanding the importance of belowground partners of crops are necessary to create sustainable agricultural systems^162–165^.

## Supplementary information

Supplementary information

Dataset 1

## Data Availability

We provide original data as a supplementary data file (Dataset 1): Supplement_original_data.xlsx
