# Supplementary material for: Drought modulates interactions between arbuscular mycorrhizal fungal diversity and barley genotype diversity
Source: Sci Rep. 2019 Jul 4;9:9650. doi: 10.1038/s41598-019-45702-1 (PMC6609766; doi:10.1038/s41598-019-45702-1)
Supplement: Supplementary file 1 — Supplementary information [file 41598_2019_45702_MOESM1_ESM.docx]

**Drought modulates interactions between arbuscular mycorrhizal fungal diversity and barley genotype diversity**

Agnieszka Sendek, Canan Karakoç, Cameron Wagg, Jara Domínguez-Begines, Gabriela Martucci do Couto, Marcel G.A. van der Heijden, Ali Ahmad Naz, Alfred Lochner, Antonis Chatzinotas, Stefan Klotz, Lorena Gómez-Aparicio, Nico Eisenhauer

SUPPORTING INFORMATION


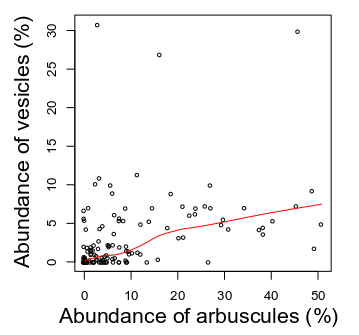


**Fig. S1.** Correlation between abundance of vesicles and arbuscules, represented as Spearman’s rank correlation coefficient (ρ) ρ=0,44, p<0,001.


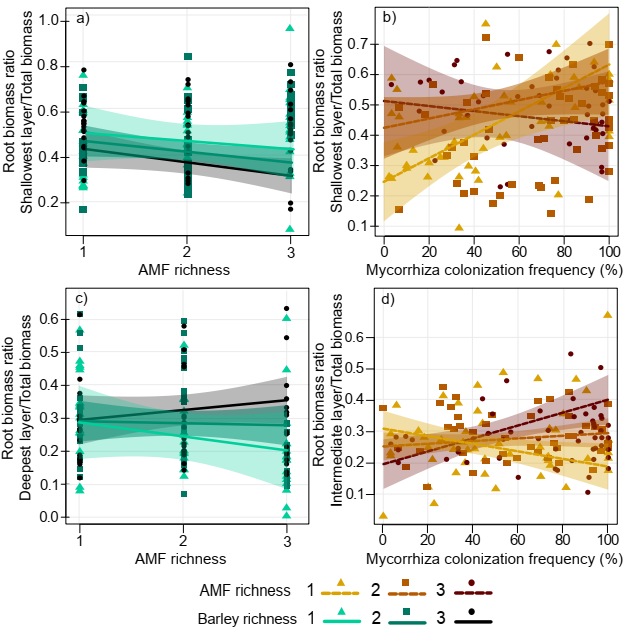


**Fig. S2.** Interactive effects of treatments on root distribution in three soil depths. Panel (a) shows the effects of barley richness and AMF richness on the root biomass in the shallowest soil layer. Panel (b) shows the effect of AMF richness and mycorrhiza colonization frequency on root biomass in the shallowest soil layer. Panel (c) shows the effect of barley richness and AMF richness on root biomass in the deepest soil layer. Panel (d) shows the effect of AMF richness and mycorrhiza colonization frequency on root biomass in the intermediate soil layer. Lines represent fitted values with 95% confidence intervals.

**
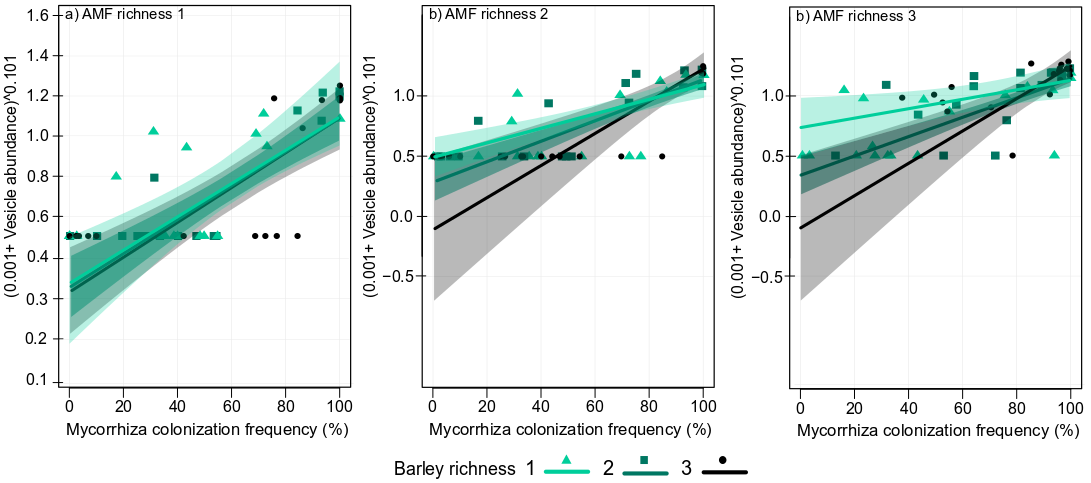
**

**Fig. S3.** Interactive effects of mycorrhiza colonization frequency, barley richness and AMF richness on the vesicle abundance. Panels (a-c) show levels of AMF richness. Lines represent fitted values with 95% confidence intervals.


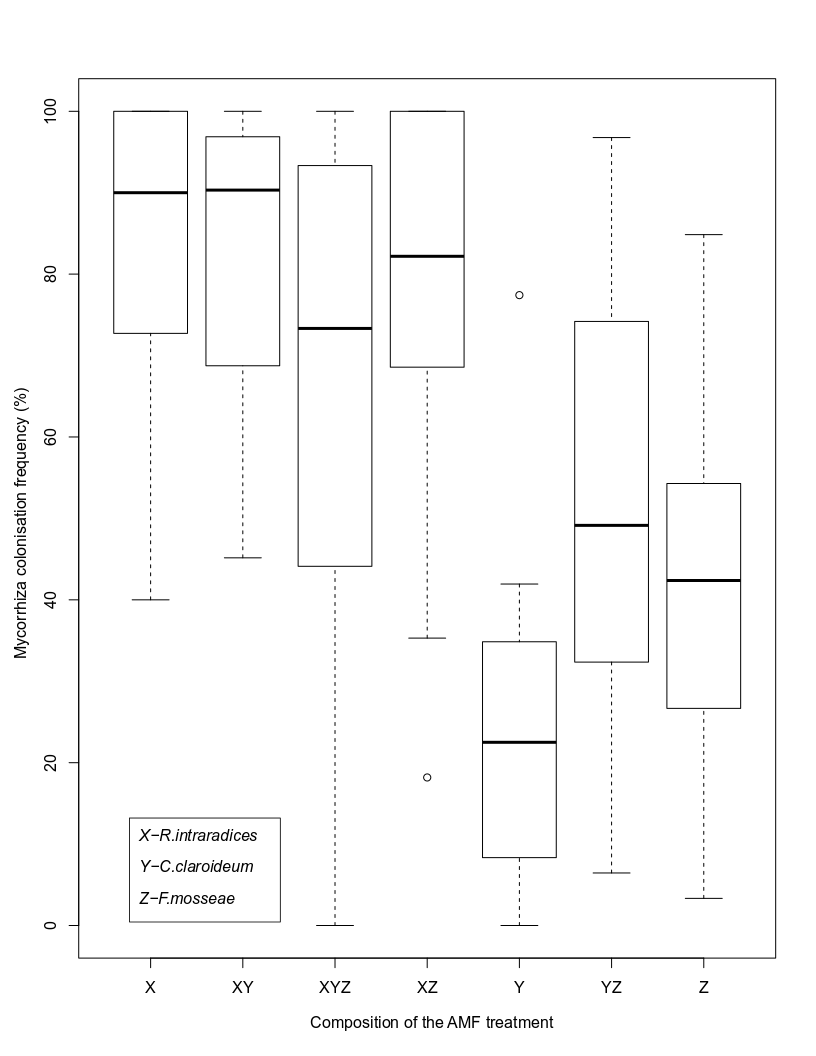


**Fig. S4.** Mycorrhiza colonization frequency of different AMF treatments used in the experiment.

Supplementary note **S1.**

Two weeks before the start of the experiment, we established four pots with a combination of three barley genotypes and three AMF species (highest levels of diversities). The pots were inoculated in the same way as it was done in the main experiment. Two of these pots obtained a proposed ambient treatment (initially 50 ml of water, Table S3), while two others were watered with a half of this amount. After we observed the signs of water deficiency (loss of turgor, yellowing of leaf tissue, premature leaf senescence) in the ambient trail pots, we increased the amount of water. Similarly, when we observed lags in water drainage, we reduced amount of water (Table S3). Based on the performance of barley in the pre-experimental trial, we determined the minimal amount of water that was needed to maintain good performance of plants, and adopted it as the ambient treatment for the main experiment. Consequently, every change of the amount of water used during the experiment was based on the trial.

**Table S1.** Nitrogen, carbon and pH values, measured in 11 randomly selected pots at the beginning of the experiment.

| Name | Nitrogen (%) | Carbon (%) | pH |
| --- | --- | --- | --- |
| Pot 5 | 0.13 | 1.71 | NA |
| Pot 9 | 0.13 | 1.77 | 7.50 |
| Pot 23 | 0.13 | 1.73 | 7.42 |
| Pot 32 | 0.13 | 1.68 | 7.40 |
| Pot 48 | 0.13 | 1.69 | 7.50 |
| Pot 57 | 0.13 | 1.67 | 7.47 |
| Pot 112 | 0.13 | 1.65 | 7.51 |
| Pot 99 | 0.13 | 1.62 | 7.48 |
| Pot 120 | 0.13 | 1.65 | 7.50 |
| Pot 122 | 0.13 | 1.70 | 7.47 |
| Pot 143 | 0.13 | 1.71 | 7.50 |

**Table S2.** Amount of water used for single watering microcosms during each week of the experiment duration.

| Time | Water amount (ml) | |
| --- | --- | --- |
|  | Ambient | Control |
| Week 1 | 50 | 25 |
| Week 2 | 50 | 25 |
| Week 3 | 50 | 25 |
| Week 4 | 50 | 25 |
| Week 5 | 50 | 25 |
| Week 6 | 80 | 40 |
| Week 7 | 120 | 60 |
| Week 8 | 120 | 60 |
| Week 9 | 140 | 70 |
| Week 10 | 160 | 80 |
| Week 11 | 160 | 80 |
| Week 12 | 140 | 70 |
| Week 13 | 140 | 70 |
| Week 14 | 120 | 60 |
| Week 15 | 100 | 50 |
| Week 16 | 60 | 30 |
| Week 17 | 40 | 20 |

**Table S3.** Test statistics of the linear models used to explore effects of drought and richness treatments on barley performance. Barley richness and AMF richness were treated as linear effects. Degrees of freedom (D.f.), values of F statistic (F) and p values (p) are presented for each main effect and interactions up to the 3rd order. Significant p values (p<0.05) are highlighted in bold. Root layers represent root biomass allocation to layers, starting from the shallowest one to the deepest one. Barley and AMF richness were treated as factor.

| Explanatory  variables | Seed mass | | | | | Shoot biomass | | | | | | Root biomass | | | | Shallowest root layer | | | | Intermediate root layer | | | | Deepest  root layer | | |
| --- | --- | --- | --- | --- | --- | --- | --- | --- | --- | --- | --- | --- | --- | --- | --- | --- | --- | --- | --- | --- | --- | --- | --- | --- | --- | --- |
|  | D.f. | | F | p | | F | | | P | F | | | | p | F | | | p | F | | | p | F | | | p |
| Drought | 1 | | 11.16 | | **0.001** | 34.55 | | **<0.001** | | | 42.71 | | | **<0.001** | 13.52 | | | **<0.001** | 0.13 | | | 0.71 | 13.79 | | | **<0.001** |
| Barley richness | 2 | | 0.32 | | 0.73 | 0.96 | | 0.39 | | | 6.67 | | | **0.002** | 1.88 | | | 0.15 | 0.52 | | | 0.59 | 1.11 | | | 0.33 |
| AMF richness | 2 | | 0.99 | | 0.37 | 2.09 | | 0.13 | | | 2.99 | | | 0.05 | 3.00 | | | 0.05 | 1.57 | | | 0.21 | 7.68 | | | **0.007** |
| Colonization frequency | 1 | | 0.01 | | 0.93 | 0.37 | | 0.55 | | | 13.05 | | | **<0.001** | 1.59 | | | 0.20 | 2.20 | | | 0.14 | 6.01 | | | **0.01** |
| Drought: barley richness | 2 | | 0.32 | | 0.72 | 2.25 | | 0.11 | | | 1.88 | | | 0.16 | 0.95 | | | 0.39 | 0.11 | | | 0.89 | 0.72 | | | 0.48 |
| Drought: AMF richness | 2 | | 0.07 | | 0.93 | 1.45 | | 0.24 | | | 2.26 | | | 0.11 | 1.55 | | | 0.21 | 0.30 | | | 0.73 | 0.95 | | | 0.38 |
| Drought: colonization frequency | 1 | | 0.94 | | 0.33 | 4.31 | | **0.04** | | | 3.34 | | | 0.07 | 2.80 | | | 0.09 | 0.38 | | | 0.54 | 1.86 | | | 0.17 |
| Barely richness: AMF richness | 4 | | 0.22 | | 0.93 | 0.37 | | 0.83 | | | 1.23 | | | 0.30 | 3.14 | | | **0.010** | 0.44 | | | 0.77 | 3.07 | | | **0.010** |
| Barely richness: colonization frequency | 2 | | 1.35 | | 0.26 | 1.27 | | 0.28 | | | 1.74 | | | 0.18 | 2.81 | | | 0.06 | 0.98 | | | 0.37 | 1.19 | | | 0.30 |
| AMF richness: colonization frequency | 2 | | 0.12 | | 0.82 | 0.10 | | 0.90 | | | 2.78 | | | 0.07 | 5.25 | | | **0.006** | 6.83 | | | **0.001** | 0.48 | | | 0.61 |
| Drought: barley richness: AMF richness | 4 | | 0.95 | | 0.44 | 0.94 | | 0.44 | | | 1.13 | | | 0.34 | 1.03 | | | 0.39 | 1.29 | | | 0.27 | 0.29 | | | 0.88 |
| Drought: barley richness: colonization frequency | 2 | | 1.99 | | 0.14 | 1.82 | | 0.17 | | | 0.14 | | | 0.87 | 0.05 | | | 0.94 | 0.16 | | | 0.85 | 0.03 | | | 0.96 |
| Drought: AMF richness: colonization frequency | 2 | | 1.27 | | 0.28 | 2.40 | | 0.09 | | | 0.71 | | | 0.49 | 0.11 | | | 0.89 | 0.59 | | | 0.56 | 0.09 | | | 0.91 |
| Barley richness: AMF richness: colonization frequency | 4 | | 1.96 | | 0.11 | 0.31 | | 0.87 | | | 1.55 | | | 0.19 | 0.98 | | | 0.42 | 0.44 | | | 0.77 | 1.04 | | | 0.38 |
| Adjusted R^2^ | | 0.04292 | | | | | 0.2171 | | | | | | 0.3553 | | | | 0.2064 | | | | 0.01683 | | | | 0.1805 | |
| No. observations | | 149 | | | | | 143 | | | | | | 150 | | | | 150 | | | | 150 | | | | 150 | |

**Table S4.** Test statistics of the linear models used to explore the effects of drought and richness treatments on AMF performance. The table shows degrees of freedom (D.f.), values of F statistics (F) and p values (p) for each main effect and interactions up to the 3rd order. Significant p values (p<0.05) are highlighted in bold. Symbol X stands for the significance of the main effects and interactions which could not be calculated when mycorrhiza colonization frequency was used as the response variable. Barley and AMF richness were treated as factor.

| Explanatory variables |  | Arbuscules | | Vesicles | | Myc. Frequency | |
| --- | --- | --- | --- | --- | --- | --- | --- |
|  | D.f. | F | p | F | p | F | p |
| Drought | 1 | 27.17 | **<0.001** | 16.93 | **<0.001** | 28.80 | **<0.001** |
| Barley richness | 2 | 17.12 | **<0.001** | 3.75 | **0.020** | 3.88 | **0.02** |
| AMF richness | 2 | 11.75 | **<0.001** | 20.31 | **<0.001** | 8.25 | **<0.001** |
| Colonization frequency | 1 | 106.78 | **<0.001** | 125.33 | **<0.001** | X | X |
| Drought: barley richness | 2 | 0.12 | 0.89 | 0.57 | 0.56 | 0.15 | 0.86 |
| Drought: AMF richness | 2 | 0.94 | 0.39 | 1.12 | 0.32 | 1.57 | 0.21 |
| Drought: colonization frequency | 1 | 0.77 | 0.38 | 0.22 | 0.64 | X | X |
| Barley richness: AMF richness | 4 | 2.63 | **0.04** | 0.64 | 0.63 | 1.81 | 0.13 |
| Barley richness: colonization frequency | 2 | 1.20 | 0.31 | 1.43 | 0.24 | X | X |
| AMF richness: colonization frequency | 2 | 0.03 | 0.97 | 1.54 | 0.21 | X | X |
| Drought: barely richness: AMF richness | 4 | 2.81 | **0.03** | 2.31 | 0.06 | 0.56 | 0.69 |
| Drought: barley richness: colonization frequency | 2 | 0.96 | 0.38 | 1.38 | 0.25 | X | X |
| Drought: AMF richness: colonization frequency | 2 | 0.95 | 0.39 | 0.37 | 0.68 | X | X |
| Barley richness: AMF richness: colonization frequency | 4 | 1.18 | 0.32 | 0.75 | 0.55 | X | X |
| Adjusted R^2^ | 0.5557 | | | 0.544 | | 0.2409 | |
| No. observations | 158 | | | 158 | | 158 | |

**Table S5.** Details of the structural equation models selection. Values of χ ^2^degrees of freedom (D.f.) as well as BIC are given for initial and most parsimonious models, for all response variables.

| Response variable | Full model | | | | Most parsimonious model | | | |
| --- | --- | --- | --- | --- | --- | --- | --- | --- |
|  | χ ^2^ | D.f. | p | BIC | χ ^2^ | D.f. | p | BIC |
| Seed mass | 0.01 | 3 | 0.9 | 2265.262 | 2.767 | 6 | 0.8 | 2252.753 |
| Aboveground biomass | 0 | 3 | 0.9 | 2233.004 | 2.398 | 6 | 0.8 | 2220.136 |
| Root biomass | 0.11 | 3 | 0.9 | 2240.337 | 1.118 | 4 | 0.8 | 2236.536 |

**Supplementary data:** Supplement_original_data.xlsx (Dataset 1) contains the original data collected during the experiment. Abbreviations used represent:

COMP_A – Barley composition (A – cultivar ‘Scarlett’ (*H. vulgare*), B – ISR42-8 (*H. vulgare*), C – *H. vulgare* ssp. s*pontaneum*),

COMP_B – AMF composition (X *–* R. *intraradices, Y – C. claroideum, Z –F. mosseae*),

DROUGHT – Water treatments: drought (1), ambient (0),

DIV_A – Barley interspecific diversity (1-3 genotypes),

DIV_B – AMF species diversity (1-3 species),

ROOT_X – Root mass on the shallowest root layer (g),

ROOT_Y – Root mass on the intermediate root layer (g),

ROOT_Z – Root mass on the deepest soil layer (g),

ROOT_SUM – Root biomass (g),

SEED_MASS – Seed biomass (g),

ABOVE_MASS – Aboveground biomass (g),

FULL_MASS – Plant biomass (g),

F – Mycorrhiza colonization frequency (%),

A – Arbuscule abundance (%),

V – Vescile abundance (%).
